# Supplementary material for: Gastrointestinal Involvement in Dermatomyositis
Source: Diagnostics (Basel). 2022 May 11;12(5):1200. doi: 10.3390/diagnostics12051200 (PMC9139828; doi:10.3390/diagnostics12051200)
Supplement: Supplementary file 1 [file diagnostics-12-01200-s001.zip › diagnostics-1647893-supplementary.pdf]

Table S1. Characteristics of patients with DM and severe GI compromise from our unit at the Hospital Clínic de Barcelona and from the literature review. PubMed/Medline from April 1990 to April 2019. ATB: antibiothic; AZA: azatioprine; CYC: cyclophosphamide; CyA: cyclosporine; F: female; GI: gastrointestinal; IFX: infliximab; IGIV: intravenous inmunoglobulines; M: male; (m): month; MPDN: methylprednisolone; MTX: methotrexate; N: no; NA: not available; PDN: prednisone; PPI: proton-pump inhibitor; Sx: surgery; Y: yes.

Reference	Date of Publication	Age	Sex	Age onset	Time from DM dx to onset of GI disease (m)	Disease activity			GI symptoms	Endoscopic Appearance	Histopathology	Diagnosis	Treatment	Outcome
						Muscle	Skin	Others						
Swenson	1968	54	F	34	240	NA	Y	N	Abdominal distention	.	.	Colonic distention	Sx	Alive
Oliveros	1973	12	F	9	36	Y	N	Y	Abdominal pain	.	.	Pneumatosis intestinalis	MPDN + MTX	Alive
Fischer	1978	8	F	8	9	Y	N	NA	Abdominal pain + fever + vomiting + diarrhea	.	Arterioles of muscle, nerve and GI tract with prominent endothelium and medial hypertrophy	Pneumatosis intestinalis	MPDN + MTX ATB	Death
Magill	1984	11	F	11	3	NA	NA	NA	Abdominal pain + fever + vomiting	.	.	Duodenum perforation	Sx + PDN	Alive
Magill	1984	15	M	13	0,5	NA	NA	NA	Abdominal pain + fever + vomiting	.	.	Duodenum perforation	Sx	Death
Schullinger	1985	6	F	6	10	Y	Y	NA	Abdominal pain	.	.	Duodenum perforation	Sx	Alive
Schullinger	1985	10	M	9	13	NA	NA	NA	Abdominal pain	.	.	Duodenum perforation	MPDN + Sx	Alive
Downey	1988	9	M	9	7	NA	NA	NA	Abdominal pain	Esophageal perforation	.	Esophageal, duodenal and colonic perforation	Sx + PDN	Alive
Downey	1988	6	F	6	5	Y	Y	N	Abdominal pain + fever	.	.	Duodenum perforation	Sx	Death
Downey	1988	5	F	5	3	Y	Y	NA	Abdominal pain + pararectal mass	.	.	Colonic perforation	Sx + PDN	Alive

Downey	1988	8	F	8	6	Y	Y	N	Abdominal pain	.	.	Duodenum perforation	Sx	Alive
Wan-Yu Lin	1995	66	M	66	12	Y	Y	Y	(Acute) Abdominal pain	.	.	Diverticular-like lesion with colon perforation	Sx	Death
Wakamatsu	1995	41	F	44	120	Y	Y	NA	Abdominal pain + distention	.	.	Pneumatosis intestinalis	.	Death
Dougenis	1996	57	F	57	6	Y	Y	Y	Abdominal pain + dysphagia + regurgitation	.	Necrosis and hemorrhage of esophagus and thinned muscle layer due to due to acute inflammation	Esophageal perforation	Sx	Alive
Stefanski	1998	7	F	7	3	NA	NA	NA	Abdominal pain	.	.	Pneumatosis intestinalis	Conservative	.
Takeda	1998	13	F	13	7	Y	Y	Y	.	.	Vessels occluded by fibrin thrombi, intimal hyperplasia and inflammatory infiltrates	Esophageal and intestinal perforation	Sx + CFM + Plasma Exchange	Death
Eshraghi	1998	24	F	24	3	NA	NA	NA	Abdominal tenderness	Erosive esophagitis, gastric/duodenal ulcers, pyloric perforation	Ischemia caused by lymphocytic vasculitis and obliterative endarteritis	Duodenal and gastric perforation	Sx + PDN + IVIG + CFM	Alive
Laskin	1999	11	M	4	60	NA	NA	NA	Abdominal pain + fever + vomiting + diarrhea	.	.	Dilated athonic esophagus	PDN + AZA + MTX	Alive
Morris-Stiff	1999	61	F	60	6	Y	Y	NA	Vomiting + weight loss	Atrophic gastritis	Pneumatosis cystoides intestinalis with a giant reaction around the gas-filed cysts	Pneumatosis intestinalis	Conservative	Alive
Wang	2001	4	M	4	2	Y	Y	Y	Abdominal pain + bile-stained vomiting	.	Perforation of the ischemic ulcerated duodenal	Duodenum perforation	Sx + MPDN	Alive

											mucosa with mononuclear cell infiltration			
Marie	2001	18	M	18	4	Y	Y	Y	Abdominal pain + gastrointestinal bleeding	Small ulcerations in stomach and duodenum	Vasculitis	GI bleeding due to vasculitis	IVIG --> PDN + MTX	Alive
Selva	2004	58	F	58	8	Y	Y	NA	Fever	.	.	Pneumatosis intestinalis	IFX + PDN + CYC + MTX	Alive
Nosho	2005	69	F	59	120	Y	Y	NA	Fever + abdominal distention	Polypoid lesions like submucosal tumors in ileum		Pneumatosis cystoides intestinalis	Conservative + Hiperoxia	Alive
Chen	2005	21	F	21	8	Y	Y	N	Abdominal distention	.	Vasculitis	Vasculitis related-colon ischemia		Death
Tweezer	2006	19	F	19	1	NA	NA	NA	Abdominal pain + diarrhea	Stomach erosions	Inflammation of antral mucosa with prominent vascular ectasia	Enteritis	IVIG	Death
Tweezer	2006	30	F	30	3	Y	Y	NA	Abdominal pain + gastrointestinal bleeding + diarrhea	Erosions, petechiae and fresh blood in colon	Diffuse mucosal and submucosal inflammation + prominent vascular ectasia	Pancolitis	IVIG + PDN	Death
Tweezer	2006	46	F	46	7	Y	Y	N	Abdominal pain + diarrhea	Erosions, ulcers and fresh blood in colon	Diffuse mucosal and submucosal inflammation + prominent vascular ectasia	Enteritis and colitis	MPDN	Alive
Mamyrova	2007	15	F	11	4	Y	Y	N	Abdominal pain + bile-stained vomiting	Esophagitis + shallow ulceration at g-e junction	Minimal inflammation in the submucosa, abnormally dilated vessels in submucosa	Enterocolitis	PDN + AZA --> IVIG	Alive
Mamyrova	2007	13	F	11	25	Y	Y	N	Abdominal pain + constipation	.	Mucosal edema without evidence of mucosal ulceration, narrowing or complete	Duodenum and colonic perforation	Sx + MPDN + CFM + IVIG	.

											occlusion of multiple small and medium arteries			
Morita	2007	19	M	19	6	NA	NA	NA	Abdominal pain	Ulcerations in duodenum	.	Duodenum perforation	Sx + PDN	Alive
Al-Haddad	2007	56	F	53	8	NA	Y	NA	Gastrointestinal bleeding	Esophagic diverticula bleeding	.	Esophageal diverticula	Conservative + PPI	Alive
Saito	2007	53	F	49	48	Y	Y	Y	Dysphagia	.	.	Pneumatosis intestinalis	Conservative + hiperoxia	.
Sibidian	2008	53	F	51	240	NA	NA	NA	Abdominal pain	.	.	Pneumatosis intestinalis	.	Death
Xiao	2008	30	F	22	96	N	Y	Y	Abdominal distention + diarrhea + weight loss	.	.	Pneumatosis intestinalis	KCl 10% + paraffin	Alive
Berard	2010	8	F	8	1	Y	Y	Y	Abdominal distention	.	.	Pneumatosis intestinalis	Conservative	Alive
Sagara	2012	59	F	56	360	Y	Y	Y	Abdominal distention	.	.	Pneumatosis intestinalis	Conservative	Alive
Sagara	2012	41	F	21	240	Y	Y	Y	Abdominal pain + peristaltism	.	Disrupted muscularis propia	Pneumatosis intestinalis	Sx	Death
Lioger	2012	67	F	66	12	Y	Y	N	Diarrhea	.	.	Vasculitis related-colon ischemia	Sx	Alive
Zarbalian	2013	51	F	49	24	NA	NA	NA	Normal	.	Pneumatosis cystoides intestinalis without inflamation	Pneumatosis intestinalis	Sx	Alive
Singh	2014	10	F	10	1	Y	Y	NA	Gastrointestinal bleeding	.	.	GI bleeding	.	Death
Singh	2014	11	M	11	108	N	Y	N	Abdominal pain	.	.	Intestinal perforation	.	Death
Burgin	2014	32	M	32	6	Y	Y	NA	Abdominal pain + fever + diarrhea	.	Disruption of crypt architecture	Malignant atrophic papulosis (Degos' disease)	ECULIZUMAB	Death
Our	2015	10	F	8	24	NA	NA	N	Refractary abdominal pain + oral intolerance	Superficial erosions in esophagus, stomach and duodenum (1st)	Vasculitis	Duodenum perforation	PDN + CYC + cloroquine + IVIG	Death

Our	2015	68	F	68	6	Y	Y	N	Abdominal pain + gastrointestinal bleeding + diarrhea	Superficial erosions in esophagus, stomach and duodenum (2–3º)	Wall inner muscular atrophy with fibrosis and edema	Esophageal perforation	Sx --> PDN + IVIG	Death
Our	2015	46	F	15	372	Y	Y	Y	Abdominal pain + gastrointestinal bleeding	Multiple ulcers in duodenum	Duodenitis with fibrosis of lamina propria and vascular ectasia	Duodenum perforation	MPDN + Plasma Exchange + IVIG --> AZA	Alive
Selva-O'Callaghan	2015	34	F	31	8	Y	N	N	Abdominal pain+ aphagya+ hematochezia	Antral ulcer + hypervascular colon mucosa with fibrin-covered ulcer	Chronic inflammation, capillary ectasia with vascular congestion	Gastric/colonic ulcers	MPDN + CYC + IVIG	Alive
Kibbi	2016	63	F	63	0	Y	Y	Y	Abdominal pain + bile-stained vomiting	Multiple ulcers + perforation of transverse colon	Vasculitis	Colonic perforation	PDN + IGIV--> Sx	Alive
Reanne Ju	2018	46	F	.	.	NA	NA	NA	Dysphagia+ tarry stool	Duodenal ulcers and stigmata of recent bleeding	Chronic duodenitis	Aortoesophageal fistula	Embolization	Death

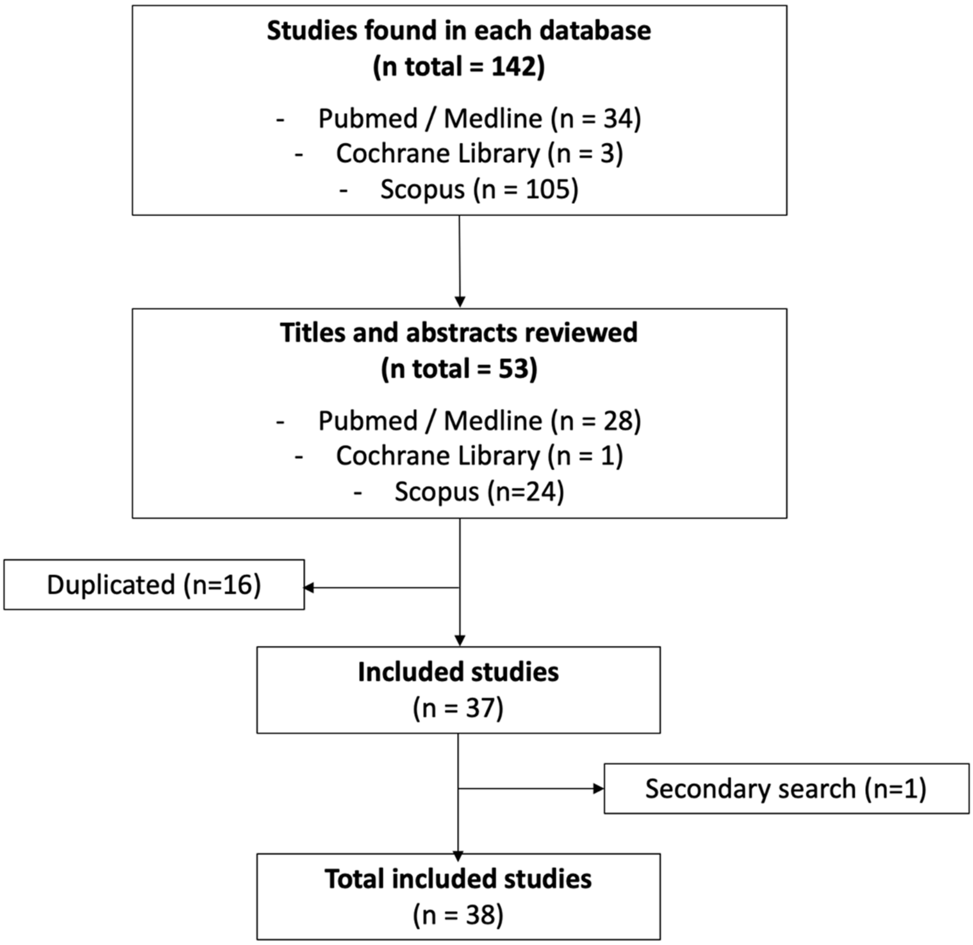

Figure S1. Flow diagram of studies selected included.
